# A Rapid Nucleic Acid Visualization Assay for Infectious Bovine Rhinotracheitis Virus That Targets the TK Gene

**DOI:** 10.1128/spectrum.01859-23

**Published:** 2023-06-29

**Authors:** Ruijia Wang, Pei Huang, Zanheng Huang, Yuanyuan Zhang, Meihui Liu, Kaikai Jin, Jiaying Lu, Yuanyuan Li, Hualei Wang, Haili Zhang

**Affiliations:** a State Key Laboratory for Diagnosis and Treatment of Severe Zoonotic Infectious Diseases, Key Laboratory for Zoonosis Research of the Ministry of Education, Institute of Zoonosis, and College of Veterinary Medicine, Jilin University, Changchun, China; Xinxiang Medical University

**Keywords:** infectious bovine rhinotracheitis virus, recombinase polymerase amplification, TK gene, on-site test

## Abstract

Infectious bovine rhinotracheitis virus (IBRV) can cause various degrees of symptoms in the respiratory system, reproductive system, and whole body of cattle. It also can lead to persistent and latent infection in cattle, posing a challenge to timely control of infectious bovine rhinotracheitis (IBR) in farms and causing large financial losses in the global cattle industry. Therefore, the goal of this study was to establish a rapid, simple, and accurate method that can detect IBRV in order to facilitate the control and eradication of IBR in cattle. We combined recombinant polymerase amplification (RPA) with a closed vertical flow visualization strip (VF) and established an RPA-VF assay that targets the thymidine kinase (TK) gene to rapidly detect IBRV. This method (reaction at 42°C for 25 min) was able to detect a minimum of 3.8 × 10^1^ copies/μL of positive plasmid and 1.09 × 10^1^ 50% tissue culture infective dose (TCID_50_) of the IBRV. This assay has high specificity for IBRV and does not cross-react with other respiratory pathogens in cattle. The concordance between the RPA-VF assay and the gold standard was 100%. In addition, this assay was also suitable for the detection of DNA from clinical samples extracted by a simple method (heating at 95°C for 5 min), which can achieve the rapid detection of clinical samples in the field. Overall, the present sensitivity, specificity, and clinical applicability assessments indicated that the RPA-VF assay we developed can be utilized as a quick and accurate on-site test for IBRV detection in farms.

**IMPORTANCE** IBRV causes different degrees of clinical symptoms in cattle and poses a great threat to the cattle industry. The infection is persistent and latent, and the elimination of IBRV in infected herds is difficult. A rapid, simple, and accurate method to detect IBRV is therefore vital to control and eradicate IBR. Combining RPA with an VF, we established an RPA-VF assay for the rapid detection of IBRV, which can complete the test of clinical samples in 35 min. The assay shows good sensitivity, specificity, and clinical applicability and can be used as an on-site test for IBRV in farms.

## INTRODUCTION

Infectious bovine rhinotracheitis (IBR) is caused by the IBR virus (IBRV), resulting in huge economic losses to the cattle industry worldwide, as well as to both domestic and wild ruminants (1, 2). IBRV, which is a herpesvirus belonging to the *Alphaherpesvirinae* subfamily and often referred to as bovine herpesvirus 1 (BoHV-1), is a highly contagious and latent virus with many negative effects on cattle respiratory diseases, reproduction, and milk production (3–6). It has also been shown that progesterone can increase the frequency of latent IBRV reactivation, which increases the risk of IBRV transmission in cows (7). Once one cow is infected with the IBRV, the virus will spread through the entire herd in a relatively short period, and the cows will carry lifelong latent infections (2, 8), all of which make it extremely difficult to eradicate the disease. New infections have emerged in recent years in some countries where IBRV infection had not previously been reported and also in countries that had previously declared that IBRV had been eradicated (9–11). A quarantine-culling strategy was adopted in a few countries (5), but it requires a huge cost and is not realistic for countries with large herds and underdeveloped economies. Furthermore, the IBR vaccine is successful in lessening the clinical effects of IBRV infection, but it does not block the establishment of the latent phase of IBRV and has the risks of abortion (4, 12).

Real-time PCR, the World Organisation for Animal Health (WOAH)-recommended method for the detection of IBRV (13), is sensitive and technically mature over PCR. However, this method depends on complicated instruments and the transport of test samples, which are time-consuming and not conducive to its widespread use in small- and medium-sized farms. Another WOAH-recommended method, serological testing, is fast and requires less equipment but may be limited in the early diagnosis of IBRV infection because it takes days or weeks to generate a detectable antibody response, leading to a lag in disease prevention and control.

**FIG 1:**
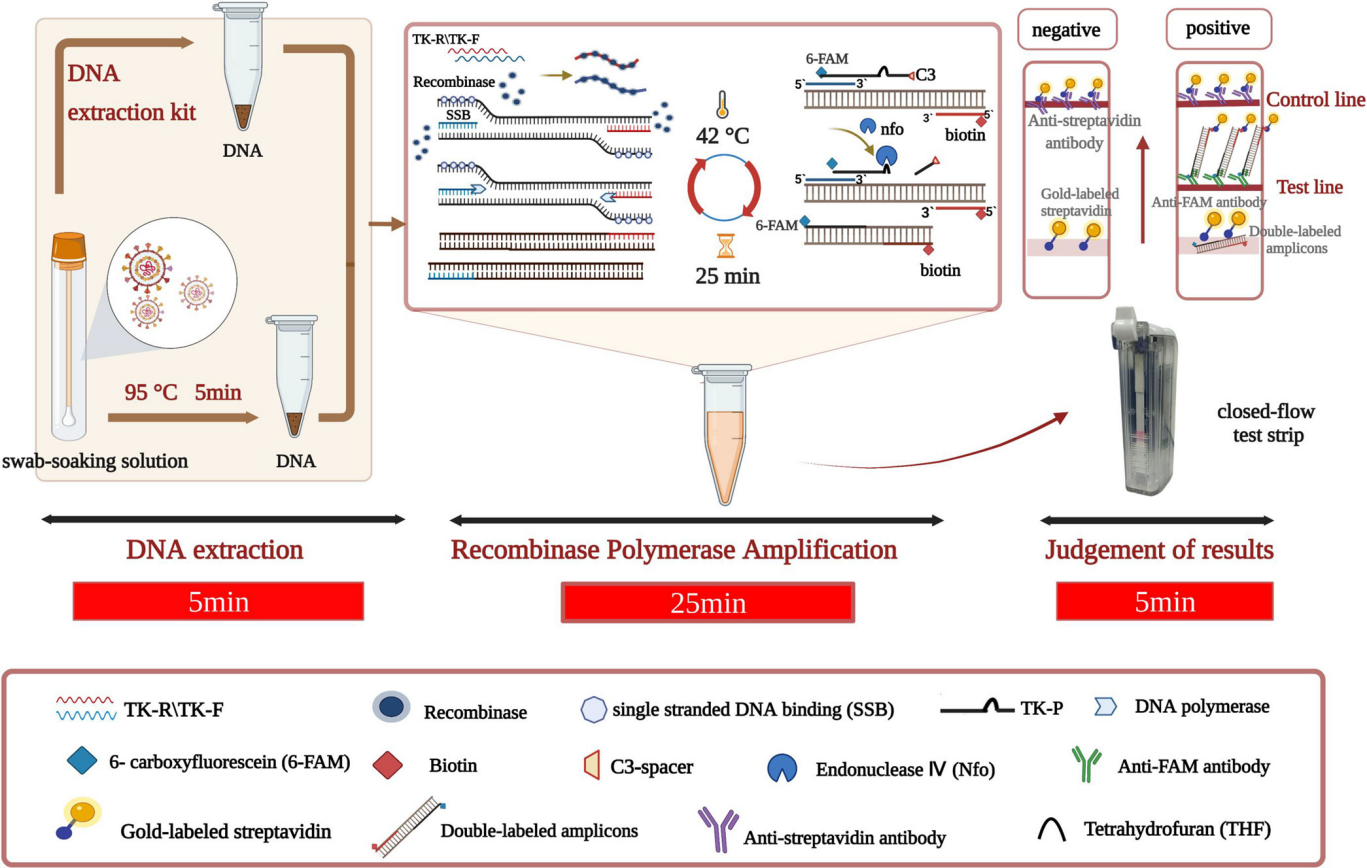
Schematic diagram of the RPA-VF assay. A DNA extraction kit (Axygen Company, China) was used to extract DNA from the swab-soaking solution. In addition, DNA of the swab-soaking solution also could be extracted quickly at 95°C for 5 min. The extracts were used as the templates, and an RPA reaction was run to form a double-labeled amplicon with 6-FAM and biotin in 25 min. The reaction tube was then placed in a VF, and then the results were judged in 5 min. In this experiment, the whole RPA-VF reaction took a total of 35 min.

Therefore, this study aims to explore a novel rapid method by combining recombinant polymerase amplification (RPA) technology with a vertical closed-flow test strip (VF). We establish here a visualized IBRV RPA-VF nucleic acid assay to provide technical support for the early and rapid diagnosis of IBR that is suitable for operation in scenarios such as early screening on cattle farms or on-site quarantine at customs (Fig. 1). The latent infection which is characteristic of IBRV in cattle means that existing rapid techniques cannot distinguish between natural infection and vaccine immunity. However, the vaccine strain widely used in some countries for the immunization of cattle carries a TK gene deletion (14). Hence, the RPA-VF rapid method we established targeting the TK gene can be used for IBRV detection in cattle and has the potential to differentiate between natural infection and vaccine immunization.

## RESULTS

### Primers and probe design.

**FIG 2:**
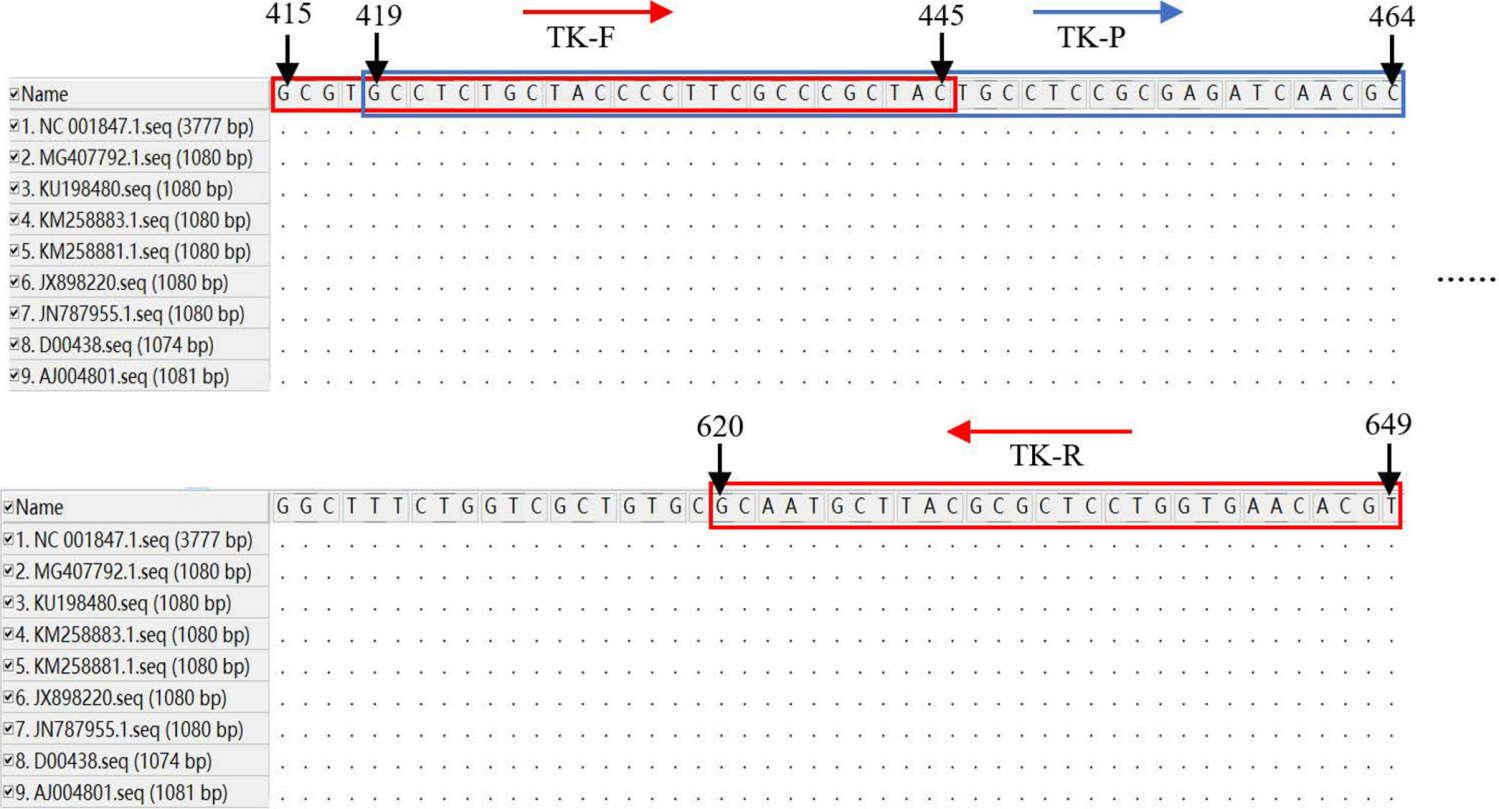
Alignment of the sequences of IBRV TK gene. A total of 9 TK gene sequences from different IBRV strains were aligned using the MEGA 11 software. TK-F and TK-R were the optimal forward primer and reverse primer for the TK gene, respectively. TK-P was the optimal probe for TK gene. The black arrow and numbers indicate the position of the primers and probe in the sequence. In addition, the region in the red box is the corresponding gene sequence of primers, and the region in the blue box is the corresponding gene sequence of the probe. The red arrows correspond to the direction of primer amplification, and the blue arrow represents the direction of probe amplification.

A total of 9 TK gene sequences from different IBRV strains were aligned using MEGA 11 software. We selected the conserved fragment of the TK gene as the target (Fig. 2) and designed six sets of primers and probes for the RPA-VF method according to the targeting sequence. The reverse primer was labeled with biotin at the 5′ end. The probe was labeled a 6-carboxyfluorescein (6-FAM) at the 5′ end, a tetrahydrofuran (THF) residue that replaced a nucleotide at 30 bp and contained a blocking group C_3_ spacer at the 3′ end so that both biotin and 6-FAM can be simultaneously integrated into a double-stranded amplicon.

### Optimization of the RPA-VF reaction conditions.

The 10-fold dilutions of the positive plasmid pBlunt-TK containing IBRV TK gene were used as a template for the RPA-VF assay. The reaction process was performed at different temperatures (45°C, 42°C, 39°C, and 37°C) for 20 min. As shown in Table 1, the detection limit for the plasmids was 3.8 × 10^2^ copies/μL at 39 to 42°C for 20 min. In the repeated experiments, 42°C was found to be the most stable temperature. Then, the reaction was performed at different times (20, 25, and 30 min) based on the above-described optimal reaction temperature, and the optimal amplification time was 25 min with a minimum detection of 3.8 × 10^1^ copies/μL pBlunt-TK DNA (Table 2).

**TABLE 1:** Optimization of the amplification temperature of the RPA-VF method for the positive plasmid pBlunt-TK

Temp (°C)	Concentration of positive plasmid pBlunt-TK (copies/μL):				
	3.8 × 10^3^	3.8 × 10^2^	3.8 × 10^1^	3.8 × 10^0^	Negative
45	0/9^*a*^	0/9	0/9	0/9	0/9
42	9/9	9/9	0/9	0/9	0/9
39	9/9	9/9	0/9	0/9	0/9
37	0/9	0/9	0/9	0/9	0/9

a Positive number/total repeats.

**TABLE 2:** Optimization of the amplification time of the RPA-VF method for the IBRV TK gene

Time (min)	Concentration of positive plasmid pBlunt-TK (copies/μL):				
	3.8 × 10^3^	3.8 × 10^2^	3.8 × 10^1^	3.8 × 10^0^	Negative
20	9/9^*a*^	9/9	0/9	0/9	0/9
25	9/9	9/9	9/9	0/9	0/9
30	9/9	9/9	0/9	0/9	0/9

a Positive number/total repeats.

### Sensitivity and specificity of the RPA-VF assay.

**FIG 3:**
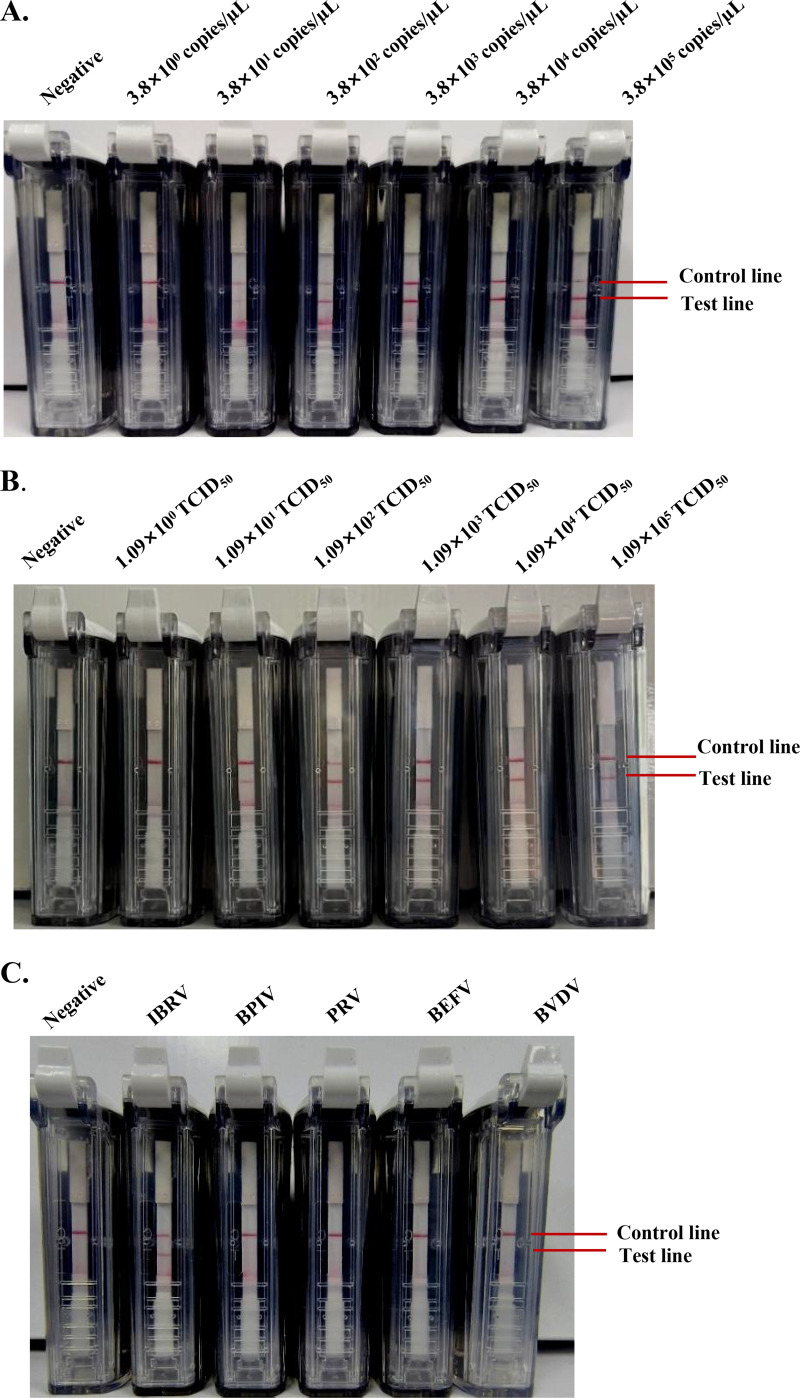
Sensitivity and specificity of the RPA-VF assay. (A) Sensitivity evaluation of the RPA-VF method. The detection limit of the RPA-VF assay was evaluated by using a 10-fold serial dilution of pBlunt-TK DNA. (B) Sensitivity evaluation of the RPA-VF method. The detection limit of the RPA-VF assay was evaluated by using a 10-fold serial dilution of IBRV. (C) Specificity evaluation of RPA-VF assay for IBRV and other viruses (bovine ephemeral fever virus [BEFV], bovine parainfluenza virus [BPIV], bovine viral diarrhea virus [BVDV], and pseudorabies virus [PRV]). Three independent experiments and three parallels in each experiment were analyzed.

The pBlunt-TK plasmids were diluted in 10-fold gradients and then utilized as the templates for the sensitivity evaluation of RPA-VF under optimized conditions. The results showed that the control line (C-line) and test line (T-line) in the VF detection device were visible when the template concentration ranged from 3.8 × 10^5^ to 3.8 × 10^1^ copies/μL, while the method gave a negative result when the concentration was ≤3.8 copies/μL (Fig. 3A).

Similarly, IBRV-infected cell cultures were diluted in 10-fold gradients to 10^5^, 10^4^, 10^3^, 10^2^, 10^1^, and 10^0^ tissue culture infective dose (TCID_50_), and then DNA was extracted as the template for virulence evaluation under optimized conditions, indicating that the RPA-VF assay could detect IBRV at a TCID_50_ of 1.09 × 10^1^ in a minimum of 25 min (Fig. 3B). The purpose of assessing levels of IBRV DNA was to evaluate the applicability of our method to viruses.

Moreover, to evaluate the specificity of the RPA-VF method, we used this method to test nucleic acids from several respiratory pathogens or herpesviruses (the cDNA of bovine viral diarrhea virus [BVDV], bovine ephemeral fever virus [BEFV], and bovine parainfluenza virus [BPIV] and the DNA of pseudorabies virus [PRV] and IBRV). The method gave a positive result for the IBRV DNA template but was negative for the other tested templates (BEFV, BPIV, BVDV, and PRV) (Fig. 3C).

### Assessment of clinical samples using the RPA-VF method.

A total of 28 nasal swabs from dairy cattle and beef cattle were assessed as positive using our IBRV RPA and showed cycle threshold (*C_T_*) values ranging from 18.14 ± 0.16 to 34.58 ± 0.28 using the real-time PCR assay. The remaining 92 nasal swabs were found to be negative using both methods. The coincidence rate between the RPA-VF assay and the gold standard was 100% (Table 3). Additionally, all samples, whether processed using a DNA extraction kit or simply inactivated at 95°C for 5 min, gave the same results of RPA detection (positive and negative results), and the detection coincidence rate for both DNA extraction methods remained at 100%.

**TABLE 3:** Comparative performances of RPA-VF and real-time RT-PCR assays for detection in clinical samples

Specimen source	Total no. of samples	Real-time PCR		RPA-VF				Concordance rate (%)
				DNA extraction kit		Pyrolysis (95°C, 5 min)		
		No. of positive samples (*C_T_* values)	No. of negative samples	No. of positive samples	No. of negative samples	No. of positive samples	No. of negative samples	
Nasal swabs of the dairy cow	72	19 (18.14 ± 0.16, 21.94 ± 0.13, 22.42 ± 0.20, 22.64 ± 0.32, 22.94 ± 0.18, 23.19 ± 0.29, 23.47 ± 0.24, 23.51 ± 0.08, 23.60 ± 0.26, 23.86 ± 0.24, 24.09 ± 0.26, 30.04 ± 0.30, 30.45 ± 0.64, 31.01 ± 0.10, 31.19 ± 0.65, 31.57 ± 0.06, 31.72 ± 0.23, 32.25 ± 0.37, 34.58 ± 0.28)	53	19	53	19	53	100
Nasal swabs of the beef cattle	48	9 (22.56 ± 0.20, 23.13 ± 0.11, 23.41 ± 0.16, 23.51 ± 0.24, 23.65 ± 0.21, 24.05 ± 0.10, 30.14 ± 0.32, 31.13 ± 0.49, 32.64 ± 0.76)	39	9	39	9	39	100

## DISCUSSION

Current control measures for IBR still focus on the early diagnosis of the disease and vaccination (8, 15). The IBR vaccine is effective in reducing the clinical impact of IBRV infection; however, it does not block the establishment of the latent phase of IBRV (6, 12). Therefore, effective methods have become a priority for the prevention and control of IBR.

In comparison to PCR and real-time PCR methods, isothermal amplification techniques are better suited for small-scale farms and on-site testing. The lowest detection limit of the loop-mediated isothermal amplification (LAMP) method constructed by researchers is 100 pg or 10 copies/μL IBRV DNA, while the results were analyzed using agarose gel electrophoresis, which cannot avoid the influence of aerosol (16, 17). Another study reported an RPA method combined with a lateral flow dipstick (LFD) assay, which had a minimum detection limit of 5 copies/μL of IBRV DNA (18). Compared to the above-described methods, although our RPA-VF method can only detect 3.8 × 10^1^ copies/μL recombinant plasmid, the VFs we used have the advantage that they are sealed independently to prevent aerosol contamination. It is worth mentioning that RPA outperforms the current LAMP approach in the detection of IBRV. LAMP analysis typically needs at least four primers, but the RPA reaction uses only two primers. Due to the high GC content of IBRV DNA, it is easier to design primers for RPA with fewer dimerization reactions.

The selection of the target gene is also a critical stage in IBRV detection. The genome of IBRV is approximately 138 kb in size and comprises several significant target genes, including gB (19), gC, gD, gE (16), TK, and UL52 (18). In this study, we selected the TK gene as a target because it is a key gene in the maintenance of persistent IBRV infection and is one of the major virulence genes (20). Furthermore, the gene sequence of IBRV TK is relatively conserved, making it a good target candidate for IBRV detection. In addition, because TK is a nonessential gene, it is one of the main target genes for developing IBR gene deletion vaccines (21–23). This means that the assay we developed can be utilized to differentiate between a naturally occurring illness and an IBRV TK deletion vaccination in cattle. Furthermore, cattle are susceptible to a variety of pathogens, of which BEFV, BPIV, BVDV, PRV, and IBRV can cause respiratory tract infections, diarrhea, and reproductive system diseases. Any method used to test clinical samples in the field must therefore have good specificity. Our assay did not show cross-reactivity with any of the above-described pathogens, demonstrating that this method is suitable for the detection of IBRV in complex clinical samples from cattle.

The shorter times and fewer steps of sample processing are more beneficial for the clinical applicability of the method. There are several available rapid methods to extract DNA from samples, of which glass beads and boiling procedures have been found to be the most effective (24). Other studies have also shown that DNA extraction can be compressed to 30 s using cellulose-based filter paper (25, 26). In this study, to shorten the sample processing steps and time, the clinical samples were inactivated simply by heating to 95°C for 5 min to quickly extract the viral DNA. Although the sensitivity of this pyrolysis DNA extraction method needs to be improved, the overall experimental time is very short (the whole reaction only took 35 min) in comparison with other methods, and the result is consistent with that from real-time PCR, suggesting that it is suitable for early clinical screening of IBRV in cattle farms. It cannot be denied that the sensitivity of our RPA-VF method needs to be improved. Therefore, our next research will focus on optimizing our RPA-VF method and improving the detection efficiency and sensitivity to create a more convenient, intuitive, and efficient point-of-care test.

## MATERIALS AND METHODS

### Animals and sampling.

Experimental animal protocols were approved by the Ethics Committee for Animal Care and Use of Jilin University (permit number SY202304007; Changchun, Jilin, China). The animals received humane care following Laboratory Animal-The Guidelines for Ethical Review of Animal Welfare (GB/T 35892-2018, China).

The nasal swabs were obtained from a dairy farm in Heilongjiang Province (72 samples) and a beef farm in Jilin Province (48 samples). Dairy cattle were lactating, aged 3 to 5 years. Beef cattle were 1 to 2 years old and asymptomatic. About 30% of the samples were taken from cows with runny nose symptoms. All the cattle were kept in a clean, ventilated, warm, and comfortable enclosure and were fed a balanced diet.

### Viruses.

IBRV and BVDV were provided by Gao Mingchun (Northeast Agricultural University), and the cDNAs of BEFV and BPIV were provided by He Hongbin (Shandong Normal University). The DNA of PRV was stored in our laboratory. All nucleic acid samples were extracted from cell-cultured viruses.

### Primers and probe design.

A total of 9 TK gene sequences from different IBRV strains (GenBank accession numbers AJ004801, D00438, JN787955, JX898220, KM258883, KM258881, KU198480, MG407792, and NC_001847) were downloaded from the National Center for Biotechnology Information database (NCBI) (https://www.ncbi.nlm.nih.gov) and were aligned using MEGA 11 software. We selected the conserved fragment of the TK gene as the target (Fig. 2) and designed six sets of primers and probes for the RPA-VF method using Primer Premier 5. All primers and probes were synthesized by Sangon Biotech Co., Ltd. (Shanghai, China).

### Construction of recombinant plasmids.

IBRV DNA was extracted from IBRV-infected cell cultures using the AxyPrep multisource genomic DNA miniprep kit (Axygen Company, China). The primers for the conserved region of the IBRV TK gene were designed by Primer Premier 5 (Table 4) and synthesized by Sangon Biotech Co., Ltd. The TK gene was then amplified with these primers using KOD FX DNA polymerase (Toyobo, Japan) and inserted into the Blunt vector (Beijing TransGen Biotech Co., Ltd., China) to construct the recombinant plasmid pBlunt-TK. The copy number of the recombinant plasmid pBlunt-TK was calculated using the formula (6.02 × 10^23^) × (ng/μL × 10^−9^)/(DNA length × 660). The most suitable set of primers (TK-F/TK-R) and probe (TK-P) was screened with PCR (Thermo Fisher Scientific, USA) and analyzed with 3% agarose gel electrophoresis (Jun Yi, China) (Table 5).

**TABLE 4:** Sequences of primers for the TK fragment (758 bp)

Target	Primer	Sequence (5′–3′）
TK	pBlunt-TK-F	GCACGGGCTGGGAAAGACAACAACGG
	pBlunt-TK-R	CACTTGAGCGCCGCGAACAGGGTGT

**TABLE 5:** Sequences of primers and probe for the IBRV RPA

Primer or probe	Location	Sequence (5′–3′）
TK-P	419–464	6-FAM-GCCTCTGCTACCCCTTCGCCCGCTACTGCCT (THF) CCGCGAGATCAACGC-C3 spacer
TK-F	415–445	GCGTGCCTCTGCTACCCCTTCGCCCGCTAC
TK-R	620–649	Biotin-ACGTGTTCACCAGGAGCGCGTAAGCATTGC

### Establishment and optimization of the RPA reaction system.

The RPA (Hangzhou ZC Bio-Sci & Tech Co. Ltd.; China) premixed reaction system (2 μM TK-F, 2 μM TK-R, 2 μM TK-P, 15.7 μL double-distilled water [ddH_2_O], and 25 μL buffer A) was formulated and transferred to lyophilized tubes containing RPA amplification enzyme and mixed thoroughly. The template (10-fold gradient dilution) was added, and then 2.5 μL buffer B (Hangzhou ZC Bio-Sci & Tech Co. Ltd.) was added to the tube cap. The tubes were wrapped with sealing film and centrifuged briefly, heated in a thermostatic apparatus to complete the RPA reaction, and then briefly placed in a 4°C refrigerator. Finally, reaction tubes were placed in the closed VF device (Ustar Biotech, Co., Ltd., China), and the results were read after 5 min. All experiments were repeated three times, and three parallels in each experiment were analyzed. A negative control was established to exclude the false-negative or false-positive results.

### Testing of clinical samples using the RPA-VF method.

DNA from clinical samples was processed in one of two ways. The first way was using a DNA extraction kit (AxyPrep multisource genomic DNA miniprep kit), and the second way was a crude extraction by heating at 95°C for 5 min. The DNA was then assessed using our RPA-VF method. Real-time PCR (the Chinese national standard [GB/T 27981-2011]) was used as the gold standard for this investigation. Each group included a negative and a positive control. Samples with a cycle threshold (*C_T_*) value of <35 were judged to be positive, and samples with a *C_T_* value of >35 and with no amplification curve in real-time PCR were judged to be negative. Each experiment was repeated three times.

### Data availability.

Further inquiries can be directed to the corresponding authors.

## References

[1] Nandi S, Kumar M, Manohar M, Chauhan RS. 2009. Bovine herpes virus infections in cattle. Anim Health Res Rev 10:85–98. doi:10.1017/S1466252309990028. 19558751

[2] Biswas S, Bandyopadhyay S, Dimri U, Patra H. 2013. Bovine herpesvirus-1 (BHV-1)–a re-emerging concern in livestock: a revisit to its biology, epidemiology, diagnosis, and prophylaxis. Vet Q 33:68–81. doi:10.1080/01652176.2013.799301. 23802762

[3] Graham DA. 2013. Bovine herpes virus-1 (BoHV-1) in cattle–a review with emphasis on reproductive impacts and the emergence of infection in Ireland and the United Kingdom. Ir Vet J 66:15. doi:10.1186/2046-0481-66-15. 23916092 PMC3750245

[4] O'Toole D, Chase CCL, Miller MM, Campen HV. 2014. Kennedy, the early sixties, and visitation by the angel of death. Vet Pathol 51:1051–1062. doi:10.1177/0300985814548515. 25362101

[5] Rodger SM, Murray J, Underwood C, Buxton D. 2007. microscopical lesions and antigen distribution in bovine fetal tissues and placentae following experimental infection with bovine herpesvirus-1 during pregnancy. J Comp Pathol 137:94–101. doi:10.1016/j.jcpa.2007.04.022. 17645893

[6] Muylkens B, Thiry J, Kirten P, Schynts F, Thiry E. 2007. Bovine herpesvirus 1 infection and infectious bovine rhinotracheitis. Vet Res 38:181–209. doi:10.1051/vetres:2006059. 17257569

[7] El-Mayet FS, Sawant L, Wijesekera N, Jones C. 2020. Progesterone increases the incidence of bovine herpesvirus 1 reactivation from latency and stimulates productive infection. Virus Res 276:197803. doi:10.1016/j.virusres.2019.197803. 31697987 PMC7068234

[8] Ackermann M, Engels M. 2006. Pro and contra IBR-eradication. Vet Microbiol 113:293–302. doi:10.1016/j.vetmic.2005.11.043. 16337098

[9] Raaperi K, Orro T, Viltrop A. 2014. Epidemiology and control of bovine herpesvirus 1 infection in Europe. Vet J 201:249–256. doi:10.1016/j.tvjl.2014.05.040. 24954868

[10] Blickenstorfer S, Engels M, Guerdat C, Saucy C, Reist M, Schwermer H, Perler L. 2010. Rhino-trachéite infectieuse bovine (IBR) dans le canton du Jura: enquête épidémiologique. Schweiz Arch Tierheilkd 152:555–560. doi:10.1024/0036-7281/a000124. 21104629

[11] Roch F-F, Conrady B. 2021. Overview of mitigation programs for cattle diseases in Austria. Front Vet Sci 8:689244. doi:10.3389/fvets.2021.689244. 34212024 PMC8239179

[12] De Brun L, Leites M, Furtado A, Campos F, Roehe P, Puentes R. 2021. Field evaluation of commercial vaccines against infectious bovine rhinotracheitis (Ibr) virus using different immunization protocols. Vaccines 9:408. doi:10.3390/vaccines9040408. 33924141 PMC8074307

[13] WOAH. 2018. OIE Terrestrial Manual 2018 as suggested by the world organization for animal health. https://www.oie.int/en/what-we-offer/expertise-network/reference-laboratories/#ui-id-3.

[14] Nettleton P, Russell G. 2017. Update on infectious bovine rhinotracheitis. InPract 39:255–272. doi:10.1136/inp.j2226.

[15] Turin L, Russo S, Poli G. 1999. BHV-1: new molecular approaches to control a common and widespread infection. Mol Med 5:261–284. doi:10.1007/BF03402063. 10390543 PMC2230419

[16] El-Kholy AA, Abdelrahman K, Soliman H. 2014. Rapid detection of BoHV-1 genomic DNA by loop-mediated isothermal amplification assay. J Virol Methods 204:81–85. doi:10.1016/j.jviromet.2014.04.011. 24769199

[17] Dong S, Feng M, Yu R, Xie C, Chen B, Li Z. 2018. Establishment and application of visual LAMP detection method of infectious bovine rhinotracheitis virus. Sheng Wu Gong Cheng Xue Bao Chin J Biotechnol 34:1587–1595. (In Chinese.). doi:10.13345/j.cjb.180008. 30394026

[18] Hou P, Wang H, Zhao G, He C, He H. 2017. Rapid detection of infectious bovine Rhinotracheitis virus using recombinase polymerase amplification assays. BMC Vet Res 13:386. doi:10.1186/s12917-017-1284-0. 29237466 PMC5729238

[19] Crannell ZA, Rohrman B, Richards-Kortum R. 2014. Equipment-free incubation of recombinase polymerase amplification reactions using body heat. PLoS One 9:e112146. doi:10.1371/journal.pone.0112146. 25372030 PMC4221156

[20] Field HJ. 1985. Resistance and latency. Br Med Bull 41:345–350. doi:10.1093/oxfordjournals.bmb.a072074. 2996685

[21] Zhang M, Fu S, Deng M, Xie Q, Xu H, Liu Z, Hu C, Chen H, Guo A. 2011. Attenuation of bovine herpesvirus type 1 by deletion of its glycoprotein G and tk genes and protection against virulent viral challenge. Vaccine 29:8943–8950. doi:10.1016/j.vaccine.2011.09.050. 21959327

[22] Ostertag-Hill C, Fang L, Izume S, Lee M, Reed A, Jin L. 2015. Differentiation of BHV-1 isolates from vaccine virus by high-resolution melting analysis. Virus Res 198:1–8. doi:10.1016/j.virusres.2014.12.012. 25556125

[23] Marawan MA, Deng M, Wang C, Chen Y, Hu C, Chen J, Chen X, Chen H, Guo A. 2021. Characterization of BoHV-1 gG-/tk-/gE- mutant in differential protein expression, virulence, and immunity. Vet Sci 8:253. doi:10.3390/vetsci8110253. 34822626 PMC8621285

[24] Tappeh KH, Hanifian H, Diba K. 2012. Comparison of four methods for DNA extraction from Echinococcus granulosus protoscoleces. Turkiye Parazitol Derg 36:100–104. doi:10.5152/tpd.2012.24. 22801915

[25] Zou Y, Mason MG, Wang Y, Wee E, Turni C, Blackall PJ, Trau M, Botella JR. 2017. Nucleic acid purification from plants, animals and microbes in under 30 seconds. PLoS Biol 15:e2003916. doi:10.1371/journal.pbio.2003916. 29161268 PMC5697807

[26] Wang Q, Shen X, Qiu T, Wu W, Li L, Wang Z, Shou H. 2021. Evaluation and application of an efficient plant DNA extraction protocol for laboratory and field testing. J Zhejiang Univ Sci B 22:99–111. doi:10.1631/jzus.B2000465. 33615751 PMC7897602

